# Supplementary material for: Cellulose Fibers Hydrophobization via a Hybrid Chemical Modification
Source: Polymers (Basel). 2019 Jul 11;11(7):1174. doi: 10.3390/polym11071174 (PMC6681115; doi:10.3390/polym11071174)
Supplement: Supplementary file 1 [file polymers-11-01174-s001.pdf]

CELLULOSE FIBRES HYDROPHOBIZATION VIA A HYBRID CHEMICAL APPRAOACH

Stefan Cichosz[a], Anna Masek[a,*]

[a] Lodz University of Technology, Institute of Polymer and Dye Technology, Faculty of Chemistry, 90-924 Lodz, Stefanowskiego 12/16, Poland
* E-mail: anna.masek@p.lodz.pl

1. Cellulose fibres sorption experiments

1.1. Method

Krüss® Tensiometer K-100 in a contact angle mode has been employed in the following experiment. During each measurement, the capillary has been filled up to 1/3 of its height and tapped 15 times in order to improve powder packing. Seven liquids were tested, as follows: distilled water, DMSO, ethanol, heptane, methanol, hexane and acetone. Not dried and dried (24 h at 100°C; Binder® oven, crystallizer 70x40 mm) cellulose fibres have been analysed.

1.2. Results

According to the data presented in **Fig. 1**, it may be observed that, generally, the sorption of various organic and inorganic solvents is higher in case of not dried cellulose fibres in comparison with the dried ones. Surprisingly, water and DMSO absorption is very low while confronted with, e.g., ethanol, heptane, hexane, acetone.

Therefore, as a polar solvent for solvent exchange experiment, ethanol has been chosen. It exhibits quite good sorption values and is less harmful than methanol. Acetone would be of the best properties, nevertheless, it is employed in chemical modification of cellulose fibres with the use of MA. Furthermore, as a non-polar solvent – hexane is the one of the most desired sorption characteristics. It is not only behaving well while cellulose is not dried, but also does it reveal high absorption for dried natural fibres.

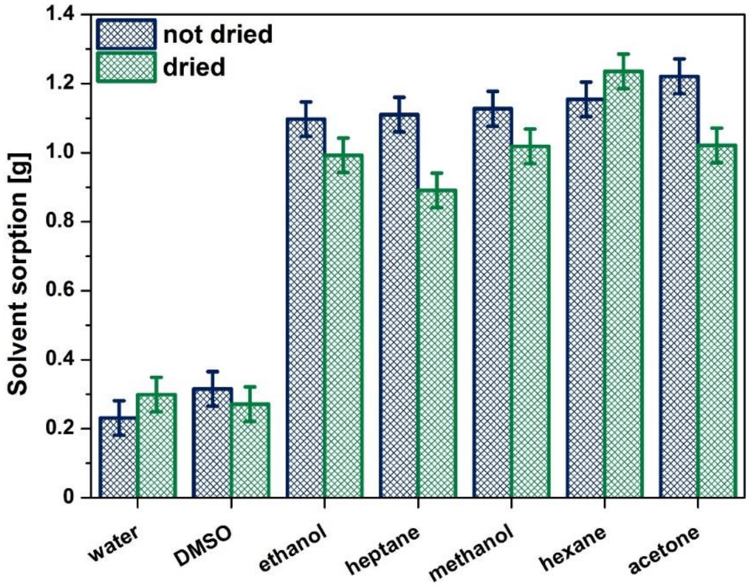

Fig. 1 Cellulose fibres sorption experiment results for various solvents.

Following experiment, proves also validity of the fact that acetone is a well-chosen liquid for discussed natural filler modification process as it exhibits the highest sorption value for not dried cellulose fibres and sufficient absorption for the dried ones.
